# Effect of Parental Migration Background on Childhood Nutrition, Physical Activity, and Body Mass Index

**DOI:** 10.1155/2014/406529

**Published:** 2014-06-01

**Authors:** Mohsen Besharat Pour, Anna Bergström, Matteo Bottai, Inger Kull, Magnus Wickman, Niclas Håkansson, Alicja Wolk, Tahereh Moradi

**Affiliations:** ^1^Institute of Environmental Medicine, Division of Epidemiology, Karolinska Institute, 17177 Stockholm, Sweden; ^2^Institute of Environmental Medicine, Unit of Biostatistics, Karolinska Institutet, 17177 Stockholm, Sweden; ^3^Department of Clinical Science and Education, Stockholm South General Hospital, Karolinska Institutet, 11883 Stockholm, Sweden; ^4^Sachs' Children and Youth Hospital, Stockholm South General Hospital, Karolinska Institutet, 11883 Stockholm, Sweden; ^5^Centre for Epidemiology and Community Medicine, Stockholm County Council, P.O. Box 1497, 17129 Stockholm, Sweden

## Abstract

*Background*. Poor nutrition, lack of physical activity, and obesity in children have important public health implications but, to date, their effects have not been studied in the growing population of children in Sweden with immigrant parents. *Methods*. We estimated the association between parental migration background and nutrition, physical activity, and weight in 8-year-old children born in Stockholm between 1994 and 1996 of immigrants and Swedish parents (*n* = 2589). Data were collected through clinical examination and questionnaires filled out by parents. Odds ratios (ORs) and 95% confidence intervals (95% CIs) were calculated using multivariable logistic regression. *Results*. Children of immigrants complied more closely with Nordic Nutrition Recommendations compared with those of Swedes (OR = 1.35, 95% CI 1.11–1.64). They had higher intake of dietary fibre, vitamins C, B6, and E, folic acid, and polyunsaturated fatty acids (omega-3 and omega-6) reflecting higher consumption of foods of plant origin, but lower intake of vitamins A and D, calcium, and iron reflecting lower consumption of dairy products. Children of immigrants had higher intake of sucrose reflecting higher consumption of sugar and sweets. Furthermore, these children had a higher risk of having low physical activity (OR = 1.31, 95% CI 1.06–1.62) and being overweight (OR = 1.33, 95% CI 1.06–1.65) compared with children of Swedish parents. The odds of having low physical activity and being overweight were even higher in children whose parents were both immigrants. A low level of parental education was associated with increased risk of low physical activity regardless of immigration background. *Conclusions*. Culturally appropriate tools to capture the diverse range of ethnic foods and other lifestyle habits are needed. Healthcare professionals should be aware of the low levels of physical activity, increased weight, and lack of consumption of some important vitamins among children of immigrants.

## 1. Introduction

Childhood physical inactivity and obesity have been increasing over the past decades and are among the most challenging public health concerns of the 21st century [1, 2]. Being overweight or obese in childhood is associated with both systemic and mental adverse consequences [3, 4]. In addition, because obese children often remain obese through adolescence and adulthood, they are at risk of metabolic disorders, cardiovascular disease, and some cancers [5] which are diseases of public health concern. Hence effective preventive programmes based on evidence from studies quantifying the risk of becoming overweight or obese in different population groups, with heterogeneous genetic backgrounds and being exposed to different environmental factors, are needed.

The aetiology of childhood overweight and obesity is a complex interaction between genetic and environmental, cultural, behavioural, and socioeconomic factors [6], all of which, apart from genetic factors, to a large extent can be influenced by immigration. Despite this, there is a lack of knowledge about the nutritional status, level of physical activity, and overweight/obesity among children of immigrant parents living in Sweden [7]. The reported studies involving the large and growing population of immigrants and their offspring in Sweden either are in adults [8–10] or confined to small groups with certain dietary habits, such as not eating breakfast or consumption of drinks with a high sugar content [10]. Moreover, almost all previous studies were conducted in first-generation immigrants, and information regarding their children is scarce.

In the present study we used data from a population-based birth cohort in Stockholm, Sweden, to assess and compare nutritional status, level of physical activity, and overweight/obesity among 8-year-old children of immigrant or Swedish-born parents.

## 2. Methods

### 2.1. Subjects

A total of 2589 children born in Stockholm between February 1994 and November 1996 in central and north-western areas of the city were included in this study. We retrieved data when the children were 8 years old from the ongoing Swedish prospective birth cohort study BAMSE (Swedish abbreviation for the “Barn/Children Allergy Milieu Stockholm Epidemiology”), with complete information on diet, physical activity, body mass index (BMI), and parental country of birth and level of education. The study area comprises different municipalities and is representative of urban and suburban districts with populations of different socioeconomic position (SEP) [11].

The baseline cohort initially included 4089 infants and was designed to study risk factors for allergic diseases during childhood. Inclusion and exclusion criteria as well as the enrollment process have been described in detail elsewhere [11]. Briefly, one of the exclusion criteria for BAMSE cohort was having a sibling already enrolled in the cohort. Then each family had contributed in the cohort with only one child and thus the children were from independent families. The baseline questionnaire (completed by parents) collected demographic data including parental country of birth and level of education when children were an average age of 2 months. Children have been followed via questionnaires (all completed by parents) at the ages of 1, 2, 4, and 8 years with 96%, 94%, 91%, and 84% response rates, respectively.

All children with completed questionnaires at 8 years of age were invited for further clinical investigation including blood sampling and anthropometric measurements. At the clinical examination, families were asked to fill out a food frequency questionnaire (FFQ) containing questions about average consumption of 98 food items and beverages during the past 12 months. In total, 2614 families (64% of the original cohort) responded to the FFQ. We excluded 25 children without information on parental country of birth. The final cohort therefore included 2589 children.

### 2.2. Ethics Statement

This study was conducted according to the principles of the Declaration of Helsinki and all procedures involving human subjects/patients were approved by the Regional Board of the Ethical Committee in Stockholm (Dnr: 2011/792-32). All parents gave their written informed consent prior to inclusion of their children in the study.

### 2.3. Data

#### 2.3.1. Nutritional Status

Data from the FFQ were transformed to nutrient intake by multiplying the frequency of consumption of each food item by its nutrient content per serving unit, using composition values obtained from the Swedish National Food Agency Database. Nutrient intakes were further adjusted for energy using the residuals method [12].

Food items were categorized into 10 dietary groups: fresh fruits, fruits products (juice, jam, marmalade, stewed fruit, and fruit soup), vegetables, cereals, potatoes, cakes and sweets, fish, meat, milk and dairy products, and eggs. The meat group was further subdivided into four groups: pork, beef or lamb, chicken and other poultry, and processed meat such as sausages and cold cuts. The reported frequencies of consumption of food items per serving unit were converted to average weekly consumption for each specific item and then added to calculate the total weekly consumption for each dietary group.

#### 2.3.2. Compliance with Nutritional Recommendations and Dietary Score

Based on compliance of intake of nutrients with guidelines using the Nordic Nutrition Recommendations of 2004 (NNR) as a proxy for a healthy diet [13], proportion of children who fully fulfil NNR for each nutrient was determined.

**Figure 1:**
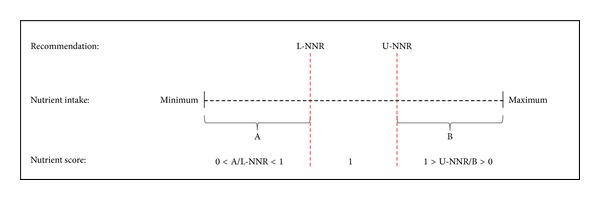
Scheme for calculation of nutrient scores, based on the Nordic Nutrition Recommendations (NNR). Nutrient intakes between the lower Nordic Nutrition Recommendations (L-NNR) and upper Nordic Nutrition Recommendations (U-NNR) scored 1. A relative score was calculated for nutrient intakes below the L-NNR using the equation A/L-NNR, and a relative score for nutrient intakes above the U-NNR was calculated by U-NNR/B, where A is the nutrient intake for a hypothetical observation below L-NNR and B is the nutrient intake for a hypothetical observation above U-NNR.

Furthermore, for each child, we calculated a dietary score indicating the overall compliance of intake of nutrients with NNR. Intake of each nutrient was scored from 0 to 1 on a continuous scale. If intake matched the recommended range or cut-off point according to the NNR, a score of 1 was assigned (full compliance with the NNR); otherwise, a relative score (between 0 and 1) was assigned based on how far the intake deviated from recommended levels (Figure 1).

Next, we classified nutrients into the following six groups: carbohydrate, fibre, fat, protein, vitamins, and minerals. The sum of the average scores in each group produced a dietary score for each child. The mean (±SD) dietary score in the entire study population was 4.82 ± 0.19 and ranged from 3.97 to 5.28 points (possible range of 0 to 6). Finally, based on the mean value, the study population was divided into two groups of high (>4.82) and low dietary score (≤4.82).

#### 2.3.3. Physical Activity

In the follow-up questionnaire at 8 years, parents were asked whether their children participated in any organized exercise or sport activity (excluding school physical education) (yes/no) and if so how often (less than once per week, 1-2, 3–5, and 6-7 times per week). Due to small numbers in some of these groups and based on existing literature, we reclassified the children into two groups: low activity (no participation or less than once per week) [14] and active (at least once per week) groups.

#### 2.3.4. Overweight and Obesity

Based on anthropometric measurements, BMI was computed for all children and interpreted according to age- and sex-specific BMI corresponding to adult cut-off values (iso-BMI) reported by Cole et al. [15]. We classified children into two categories: normal weight (iso-BMI <25 kg/m^2^) and overweight (iso-BMI ≥25 kg/m^2^). The overweight category includes obese children with iso-BMI values ≥30 kg/m^2^, unless otherwise specified as overweight and obese separately.

#### 2.3.5. Parental Country of Birth and SEP

Children were classified based on their parental country of birth: Swedish, if both parents were born in Sweden and immigrant, if at least one parent was born outside Sweden. The immigrant parent category was further subdivided into both parents; only mother; only father. We further categorized five groups of children whose parents were born in the same region of the world, based on the definitions of the United Nations Population Division [16] (Africa, Asia, Latin America, Europe excluding Sweden, and Sweden), and two mixed parental groups (mixed excluding Sweden and mixed including Sweden).

We found that the distribution of parental country of birth in our study was similar to and therefore representative of the baseline population, with regard to birth country, in BAMSE as well as the total population of Sweden in 1994. The latter comparison was made using the Swedish Total Population Register and Multigeneration Register in which data on parental country of birth is available for the entire Swedish population [17].

The highest attained level of parental education was used as an indicator of family SEP. Parental education was classified into three categories: ≤9 years (elementary school), 10–12 years (high school), and >12 years (university) of education.

### 2.4. Statistical Analysis

We used the *t*-test to compare mean consumption of dietary groups and mean intake of nutrients (macro- and micronutrients) between children of immigrant parents and of Swedish parents, and we used the chi-squared test to compare compliance with NNR among children with parental migration backgrounds and with Swedish parents. In addition, we checked characteristics of our study population with participant and nonparticipants at 8-year follow-up clinic as well as with baseline population in BAMSE regarding sex, parental education and smoking, BMI status at 4 years (the preceding clinical examination), and birth weight using *t*-test and chi-squared test, for continuous and categorical variables, respectively. Despite the large sample size, each of the *t*-tests was rechecked by nonparametric equivalent, Mann-Whitney *U* test, for taking into account departure from normality where we found complete agreement.

We used logistic regression to estimate the association between parental migration background and iso-BMI (normal versus overweight), nutritional status (high versus low dietary score), and physical activity (low versus active). The analysis was adjusted for sex and parental level of education, as a proxy for socioeconomic position, in addition to other potential risk factors such as iso-BMI, dietary score, and physical activity, when applicable. *P* values <0.05 were considered to be statistically significant. We used the Statistical Analysis System statistical software package version 9.3 (SAS Institute, Cary, NC, USA).

## 3. Results

### 3.1. Demographic Characteristics

Table 1 shows the characteristics of the study population by parental migration status, sex, dietary score, physical activity, iso-BMI, and level of parental education. There were slightly more girls than boys among children of immigrant parents.

The proportion of children with high dietary scores and the highest parental education level (>12 years) was higher among offspring of immigrant parents compared to children of Swedish parents. However, being overweight, having low physical activity, and having the lowest level of parental education (<9 years) were all more common among children of immigrant parents, particularly among children of both immigrant parents (Table 1).

The result of comparison between our study population, participant at 8-year clinic, and nonparticipants at 8-year follow-up clinic as well as with baseline population in BAMSE regarding sex, parental education and smoking, BMI status at 4 years (the preceding clinical examination), and birth weight showed no significant differences (data not shown).

### 3.2. Food Consumption

Consumption of fruit, vegetables, beef/lamb, chicken/poultry, and eggs was significantly higher among children of immigrant parents, whereas consumption of potatoes, processed meat, and milk and dairy products was lower compared to children of Swedish-born parents. There was no difference in consumption of fruit products, cereals, cakes and sweets, fish, pork, and total meat between the two groups of children (Table 2).

In the analysis stratified by sex, higher consumption of beef/lamb in children of immigrant parents was confined to girls, and lower consumption of potatoes, processed meat, and milk and dairy products was confined to boys (data not shown).

### 3.3. Nutrient Intake and Compliance with NNR Recommendations

Mean energy intake as well as intake of macronutrients including total carbohydrate, fats, and protein was similar in children of Swedish and immigrant parents (Table 3). These results were consistent in subgroups of children of immigrant mothers and/or fathers in both sexes (data not shown). However, there was a lower intake of saturated fatty acids (SFAs), vitamins A and D, riboflavin, calcium, iron, and phosphorus among offspring of immigrants whereas intake of sucrose, dietary fibre, polyunsaturated fatty acids (PUFAs) (omega-3 and omega-6) vitamins E, C, and B6, and folic acid was higher, compared with children of Swedish parents (Table 3).

Overall, compliance with NNR guidelines for nutrients such as dietary fibre, SFAs, PUFAs (omega-3), and vitamin D was high in only a small proportion of children regardless of parental country of origin (Table 4). A higher proportion of offspring of immigrants complied with these recommendations regarding dietary fibre, SFAs, and PUFAs including omega-3 while a lower proportion complied with regard to protein, vitamin A, iron, and zinc, compared with children of Swedish parents (Table 4). These findings were particularly notable among children whose parents were both immigrants (data not shown).

### 3.4. Dietary Score

Children of immigrants were more likely to have a high dietary score (i.e., higher compliance with NNR) compared with those of Swedes (OR = 1.31, 95% CI 1.08–1.58) adjusting for sex, iso-BMI, physical activity, and parental education. These results were consistent in analyses stratified by parental level of education (data not shown). In subgroups of children of immigrants, this finding was statistically significant only in children (both boys and girls) with an immigrant father (OR = 2.19, 95% CI 1.59–3.03) (Table 5). Additional stratified analysis by parental migration background revealed that children in the lower categories of parental education compared with the highest group were less likely to have a high dietary score regardless of parental birth country (data not shown).

### 3.5. Physical Activity and Overweight

The likelihood of both having low physical activity and being overweight was 30% higher among children of immigrant parents compared to those of Swedish parents, after adjusting for sex, iso-BMI, dietary score, and parental education (Table 5). These results were consistent in analyses stratified by parental level of education (data not shown). The odds ratios were the highest among offspring whose parents were both immigrants: 66% (95% CI, 18–132) and 70% (95% CI, 20–140) higher for having low physical activity and being overweight, respectively, compared with children of Swedish parents. Stratified analysis by sex revealed that girls with both immigrant parents were the most likely to be overweight, with about 2-fold increased risk compared with offspring of Swedish parents. Stratified analysis by parental migration background suggested that a low level of parental education was associated with increased risk of low physical activity in children regardless of migration background. A low level of parental education was also associated with being overweight, but only in children of Swedes (data not shown).

### 3.6. Parental Birth Region

Children of Asian parents had significantly higher odds of having low physical activity (OR = 2.19, 95% CI 1.28–3.74) and of being overweight (OR = 2.99, 95% CI 1.76–5.07) compared with children of both parents born in Sweden (Table 6). Furthermore, compared with children of two Swedish parents, low physical activity was increased approximately 2-fold in offspring of mixed non-Swedish parents (OR = 2.17, 95% CI 1.07–4.39), and children of Latin American parents had almost ten times the risk of being overweight (OR = 9.66, 95% CI 1.86–50.18). Only among children of mixed Swedish/immigrant parents was the odds ratio for high dietary score significantly higher compared with children of Swedish parents (OR = 1.44, 95% CI 1.15–1.80) (Table 6).

## 4. Discussion

In this cross-sectional study we found that offspring of immigrants complied more fully with nutritional recommendations but had a higher risk of having low physical activity and being overweight, compared with children of Swedes. The odds of having low physical activity and being overweight were even higher when both parents were born outside Sweden. We further observed that an increased risk of low physical activity was associated with a low level of parental education regardless of parental migration status. In line with recommendations, intake of SFAs was lower and intake of dietary fibre, PUFAs (omega-3 and omega-6), vitamins E, C, and B6, and folic acid was higher among offspring of immigrants whereas, contrary to guidelines, intake of vitamins A and D, calcium, and iron was lower and intake of sucrose was higher, compared with offspring of Swedes.

To our knowledge, this study is the first conducted in Sweden to explore the association between parental geographical region of birth and childhood nutrition, physical activity, and iso-BMI. Strengths of our study include the high proportion (84%) of children who answered the follow-up questionnaire at 8 years of age and the relatively high rate of participation (64%) in the clinical examination with the simultaneous use of a validated FFQ. In addition, the distribution of country of birth for immigrant parents in our study population was similar to the distribution among immigrant populations in the baseline BAMSE cohort as well as in the total Swedish population. Moreover, we observed no differences between our study population, nonparticipants in 8-year follow-up clinic, and baseline BAMSE cohort regarding sex, parental education, smoking, preceding BMI status, and birth weight. Thus, selection bias seems unlikely, but it cannot be ruled out completely. The results of our cross-sectional study are not limited by the known problem of reverse causality inherent in most cross-sectional studies, because the studied exposure (parental countries of birth) is not affected by the studied outcomes (physical activity, overweight/obesity, and nutritional status).

To our knowledge, the novel findings of the present study of “healthier” dietary habits among children of immigrants compared with children of native Swedes, including higher intake of fruits and vegetables and of protein from nonprocessed meat and eggs as well as “healthier” nutrient intake in the form of lower levels of SFAs and higher PUFA (omega-3 and omega-6) and fibre consumption, have not previously been explored. These results are, however, in contrast to the findings of a systematic review of dietary habits among adults of different ethnic groups living in Europe [18]. Nevertheless, the observed lack of compliance with NNR guidelines for dietary fibre, SFAs, PUFAs (omega-3), and vitamin D in a high proportion of children in our study, regardless of parental country of birth, warrants further investigation.

Intake of fibre is inversely associated with the risks of obesity and diabetes mellitus as well as constipation [19]. PUFAs and in particular N-3 fatty acids are considered to be important for the development of the neural system especially the brain and retina, as well as psychomotor development and mental performance [20]. It has been suggested that vitamin D deficiency increases the risk of chronic conditions such as rheumatoid arthritis, type I and II diabetes, multiple sclerosis, cardiovascular diseases, obesity, and skeletal problems [21]. The finding of lower intake of vitamin D as well as higher odds of being overweight among children of immigrants compared with those of Swedes observed in this study supports previous results showing an association between low vitamin D level and obesity and related chronic diseases such as type II diabetes and metabolic syndrome [22, 23]. Other factors, in addition to the low dietary intake of vitamin D, could be considered potential predisposing factors towards diminished ability to biotransform vitamin D from sunlight in some subgroups of offspring of immigrants, including dark skin [24] and increased body area covered by clothes for religious or cultural reasons [25].

Overall, we observed higher odds of a high dietary score among offspring of immigrants. However, among subgroups of children of immigrants, with the exception of the only father immigrant group, dietary scores were similar to those of offspring of Swedes. Because the reference values used in this study are based on the NNR, the observed relatively higher dietary score in children of immigrants could be a sign of integration in terms of Swedish dietary habits and acculturation or might be considered an influence of healthy food habits from the country of parental origin. Furthermore, the observed differences in subgroups of children might be explained by balance between influences of unhealthy dietary habits gained in the host country, which could be rapidly adopted [26], and healthy food habits from parental birth country. Assuming a more important role of mothers in children nutrition than fathers, children of Swedish mothers and immigrant fathers might benefit from synergistic effect of healthy dietary habits of both countries of mother and father and thus the observed highest odds of high dietary score, whereas healthy dietary habits of original country among children of immigrant mothers regardless of father's birth country might have been counterbalanced by adopted unhealthy dietary habits in the host country. However, there was a lack of culturally adapted questions in the FFQ, for example, regarding ethnic food items. In addition, despite the reasonable study sample size, we were not able to perform a more detailed analysis based on individual parental country of birth due to lack of statistical power.

The increased odds of low physical activity among offspring of immigrants compared with children of the host country in our study are in line with some previous findings [27]. Lack of information among immigrant parents about available facilities and opportunities for physical activity for children [28], or economic limitations, might explain the observed increased odds of low physical activity among children of immigrants in this study, especially those with both immigrant parents. Moreover, the tendency towards sedentary behaviours such as watching television and screen-based games has increased during past decades [1], which in part might explain the low physical activity observed in this study among children with a low versus a high parental level of education regardless of migration background. However, physical activity questions in the questionnaire did not correlate highly with objective measures of physical activity as well as free play, games, transport to school, school physical education, and recreational activities, the levels of which could be relatively high among children in the age group included in this study, especially in a Swedish school and neighborhood context that try to keep up children as much active as possible. Since the organized exercise has been taken as proxy for physical activity the results should be interpreted with caution.

The observed prevalence of overweight among children born in Stockholm in our study (21.5%), irrespective of parental migration background, is in line with results of the Swedish national survey in 2003 among children in the same age range [29] and with results from studies from northern, western, and central parts of Sweden as well as Stockholm, also among children in the same age range and during a similar calendar period [30–33]. Moreover, the increased risk of being overweight among offspring of immigrants in our study is in agreement with the results of studies among non-Hispanic black and Mexican American children in the USA [34], schoolchildren in Germany with foreign-born parents [14], children of Turkish and Moroccan descent in the Netherlands [35], and children of immigrants from Turkey and the former Yugoslavia in Austria [36]. A higher prevalence of several established risk factors for being overweight such as a low degree of physical activity and low parental education level might explain the observed increased odds of being overweight among offspring of immigrants, especially those with both immigrant parents. However, this finding was not affected by adjustment for these risk factors. Furthermore, departure from traditional diets among offspring of immigrants could partly explain the observed higher odds of being overweight among this group in our study. A lower rate of obesity among sub-Saharan African children who migrated to Australia has been associated with a more traditional cultural orientation including diet [37].

An association between being overweight in childhood and a low level of parental education as an indicator of low SEP, especially among children of immigrants, has been shown in some previous cohorts [38] and cross-sectional studies [14, 39] but not in a cross-sectional study in which parental employment was used as an indicator of SEP [40]. The use of a variety of indicators of SEP including parental education, parental employment, household size, or a combination of these factors might explain the observed discrepancies. Lack of such an association among children of immigrants in our study mirrors the relation between childhood obesity and socioeconomic status in developing countries [41] and might also reflect cultural attitudes towards and parental perception in some cultures of being overweight in childhood as a sign of being healthy and well nourished.

Our results support the role of parental education in physical activity and nutrition of the offspring, regardless of migration background. Health awareness and concerns among well-educated parents could explain the observed association. Nevertheless, one cannot overlook existing diversities in overweight by migration background, which might highlight the role of genetic and cultural background in developing overweight in children.

Although association of lifestyles, nutrition, and physical activity with overweight and their twisted skein of interrelations in between have been taken into account in our analysis, we lacked information on other factors such as parental age at migration, duration, and area of residence.

Given changes in dietary habits over time, the fact that our baseline data were collected about 10 years ago could be considered as a limitation, especially for children of immigrant parents in whom acculturation is an additional factor. Nevertheless, changes in dietary habits and food preference are not a short time process and might need generations to occur [18]. On the other hand, our data has been collected in context of an ongoing birth cohort (recently 16-year follow-up has been completed); then this “limitation” becomes the strength. The data provide a baseline for assessment of changes over time as well as development of overweight and obesity later on life.

Sufficient knowledge of the Swedish language was one of the criteria for inclusion in the BAMSE cohort. Our estimates would be biased if a lack of parental knowledge of Swedish language is related to low physical activity and overweight among their children. The observed association between higher prevalence of inactivity and being overweight among children of immigrant parents compared with children of Swedes would be attenuated if children of non-Swedish-speaking immigrant parents were both more inactive and overweight, as expected of those living in deprived suburbs with a high proportion of immigrants [42]. The same underestimation of the observed higher prevalence of inactivity and being overweight in children of poorly educated immigrant parents would be likely if the lack of parental knowledge of Swedish is related to their low educational level. However, poor Swedish language might, in some immigrant groups, be related to having recently arrived in Sweden rather than to poor education. But, it is more likely that the educational level would differ among recent immigrants.

## 5. Conclusion

In conclusion, the observed higher prevalence of having low levels of physical activity, being overweight, and having lower intakes of vitamins A and D, calcium, and iron reflecting lower consumption of milk and dairy products, among children of immigrant parents, warrants more focused studies with larger samples of children using culturally appropriate tools to capture the diverse range of ethnic foods and other lifestyle habits. From a public health point of view, our result calls for preventive measures especially among children where both parents are immigrants. Further preventive actions regarding dietary habit and physical activity should be directed to children of parents of low educational level regardless of parental migration background. But in the meantime, healthcare professionals should be aware of low physical activity levels, increased iso-BMI, and different nutritional habits among the children of immigrants in Sweden.

## Figures and Tables

**Table 1:** Demographic characteristics of 8-year-old children born between 1994 and 1996 in Stockholm, by parental migration status.

	Swedish	Immigrant				Total
		Any parent	Both parents	Only mother	Only father	
Number of children	2028	561	171	200	190	2589
Proportion (%)	78.3	21.7	6.6	7.7	7.3	100
Sex (%)						
Girls (*n* = 1267)	47.9	52.8	56.7	47.5	54.7	48.9
Boys (*n* = 1322)	52.1	47.2	43.3	52.5	45.3	51.1
Dietary score (%)						
Low	49.8	43.0	48.0	49.5	31.6	48.3
High	50.2	57.0	52.0	50.5	68.4	51.7
Physical activity (%)						
Low activity	22.8	28.0	33.9	22.1	29.0	24.0
Active	77.2	72.0	66.1	77.9	71.0	76.0
Iso-BMI^a^ (%)						
<25 kg/m^2^	79.7	74.2	69.0	77.0	75.8	78.5
25–30 kg/m^2^	16.7	18.5	20.5	18.5	16.8	17.1
≥30 kg/m^2^	3.6	7.3	10.5	4.5	7.4	4.4
Parental education^b^ (%)						
≤9 years	1.7	3.8	4.7	2.5	4.2	2.1
10–12 years	44.6	41.1	42.1	38.5	42.9	43.9
>12 years	53.7	55.2	53.2	59.0	52.9	54.0

^a^Sex- and age-standardized BMI corresponds to adult cut-off points.

^b^Completed years of education.

**Table 2:** Food consumption^a^ in 8-year-old children born between 1994 and 1996 in Stockholm, by parental migration status.

Food groups	Swedish		Immigrant							
			Any parent		Both parents		Only mother		Only father	
	Mean	SD	Mean	SD	Mean	SD	Mean	SD	Mean	SD
Fruits	11.1	7.7	12.4**	9.3	13.1**	8.9	11.8	9.6	12.4	9.4
Fruit products	6.0	4.6	6.3	5.5	6.3	5.4	6.3	5.5	6.2	5.7
Vegetables	17.2	10.0	18.2*	11.0	18.1	12.3	17.8	10.3	18.7	10.5
Cereals	9.7	4.3	9.4	4.5	8.9	5.2	9.7	4.4	9.6	3.9
Potatoes	5.1	2.4	4.8*	2.4	5.0	3.0	4.8*	2.1	4.8*	2.1
Cakes and sweets	9.2	4.6	9.5	5.8	10.9**	7.6	9.0	4.7	8.8	4.6
Fish	2.6	1.9	2.7	1.8	2.6	1.9	2.7	1.8	2.7	1.6
Total meat	11.0	4.9	10.9	6.0	10.7	7.6	11.2	5.2	10.8	5.2
Pork	1.0	0.8	0.9	0.8	0.9	0.9	1.0	0.8	0.9	0.8
Beef/lamb	3.6	1.7	3.9*	2.4	4.2*	3.3	3.8	1.8	3.7	1.8
Chicken/poultry	1.3	0.8	1.4**	0.8	1.5***	0.9	1.3	0.8	1.4	0.9
Processed	5.2	3.6	4.7*	4.2	4.2*	5.0	5.1	3.8	4.8	3.7
Milk and dairy products	25.2	13.1	23.1***	13.9	22.6*	13.3	23.6	14.5	23.0*	13.8
Eggs	0.8	1.0	1.0***	1.1	1.3***	1.3	1.0*	1.2	0.8	0.8

Number of children	2028		561		171		200		190	

Values given in bold are statistically significant; SD: standard deviation.

^a^Mean consumption per serving unit of food groups per week.

Mean consumption of food groups was significantly different in immigrant children compared with Swedish children (*t*-test): *P < 0.05, **P < 0.01, ***P < 0.001.

**Table 3:** Nutrient intake in 8-year-old children born between 1994 and 1996 in Stockholm, by parental migration status.

Nutrient	Swedish		Immigrant	
	Mean	SD	Mean	SD
Energy (Kj/kg)				
Girls	267.9	82.6	274.4	89.6
Boys	278.7	79.8	273.6	90.0
Macronutrients				
Carbohydrate (g/d)	253.6	20.7	254.0	23.3
Sucrose (g/d)	45.6	11.8	47.3**	13.3
Dietary fibre (g/Mj/d)	2.3	0.5	2.4***	0.5
Fat (g/d)	65.0	8.3	64.9	9.1
Cholesterol (mg/d)	245.0	49.7	246.8	54.2
Fatty acids (g/d)				
Saturated	29.2	5.1	28.7*	5.6
Monounsaturated	22.6	3.0	22.7	3.4
Polyunsaturated	8.1	1.5	8.5***	1.9
Omega-3	1.4	0.3	1.5***	0.4
Omega-6	6.5	1.3	6.8***	1.5
Protein (g/d)	75.0	9.1	74.4	10.4
Micronutrients				
Vitamins				
Vitamin A^a^ (*μ*g/d)	1111.0	369.9	1049.9***	366.6
Vitamin D (*μ*g/d)	5.3	1.6	5.1**	1.8
Vitamin E (mg/d)	7.0	1.2	7.2***	1.3
Vitamin C (mg/d)	96.8	35.9	103.3***	39.9
Thiamin (mg/d)	1.2	0.1	1.2	0.2
Riboflavin (mg/d)	2.04	0.4	1.97***	0.4
Niacin^b^ (mg/d)	27.7	3.0	28.0	3.7
Vitamin B6 (mg/d)	1.91	0.27	1.94*	0.30
Vitamin B12 (*μ*g/d)	5.7	1.5	5.6	1.7
Folic acid (*μ*g/d)	214.5	32.6	222.5***	37.6
Minerals				
Calcium (mg/d)	1239.9	345.4	1162.8***	348.6
Iron (mg/d)	10.80	1.79	10.57**	1.83
Magnesium (mg/d)	303.0	28.6	302.7	32.8
Phosphorus (mg/d)	1468.8	240.2	1431.8***	248.8
Potassium (mg/d)	3525.0	436.7	3473.6*	467.8
Selenium (*μ*g/d)	34.9	6.0	35.2	7.2
Sodium (mg/Mj/d)	334.6	44.9	335.8	52.9
Zinc (mg/d)	10.3	1.3	10.3	1.6

Number of children	2028		561	

Values given in bold are statistically significant; SD: standard deviation; Mj: mega joule; Kj: kilojoule.

^a^Vitamin A as retinol equivalent.

^b^Niacin as niacin equivalent.

Mean nutrient intake in immigrant children was significantly different (*t*-test) compared with nutrient intake in Swedish children: *P < 0.05, **P < 0.01, ***P < 0.001.

**Table 4:** Proportion of 8-year-old children born between 1994 and 1996 in Stockholm who complied with the Nordic Nutrition Recommendation (NNR), by parental migration status.

Nutrient	NNR	Fulfill NNR (%)	
		Swedish	Immigrant
Carbohydrate	50–60 %*E*	72.2	70.6
Sucrose	≤10 %*E*	58.5	55.4
Dietary fibre	≥3 g/Mj/d	7.1	11.1**
Total fat	2535 %*E*	79.8	78.1
Saturated fat	≤10 %*E*	4.7	7.3*
MUFA	10–15 %*E*	69.4	66.1
PUFA	5–10 %*E*	8.1	11.6*
Omega-3	1 %*E* ^a^	3.1	6.2***
Protein	10–20 %*E*	97.8	95.4***
Vitamins			
A	≥400 *μ*g/d	100.0	99.5***
D	≥7.5 *μ*g/d	9.4	7.5
E	≥6 mg/d	79.0	82.5
C	≥40 mg/d	98.1	98.4
Thiamin (B1)	≥0.9 mg/d	98.6	98.8
Riboflavin (B2)	≥1.1 mg/d	99.3	99.8
B6	≥1 mg/d	100.0	99.8
Folic acid	≥130 *μ*g/d	100.0	100.0
Minerals			
Calcium	≥700 mg/d	94.7	93.1
Iron	≥9 mg/d	85.1	80.6**
Magnesium	≥200 mg/d	99.9	100.0
Zinc	≥7 mg/d	99.6	98.4**

Number of children		2028	561

Values given in bold are statistically significant; %*E*: percentage of energy intake.

MUFA: monounsaturated fatty acid.

PUFA: polyunsaturated fatty acid.

^a^For calculation we considered the range of 0.5–1.5 %*E*.

Compliance with NNR in immigrant children was significantly different compared with children of Swedish parents (chi-squared test): *P < 0.05, **P < 0.01, and ***P < 0.001.

**Table 5:** Odds ratio (OR) and 95% confidence interval (CI) of dietary score, low physical activity, and overweight, by parental migration status.

Parental migration status	Sex	*n*	Dietary score^a^		Low physical activity^b^		Overweight^c^	
			OR^d^	95% CI	OR^d^	95% CI	OR^d^	95% CI
Swedish (Ref.)			1.00		1.00		1.00	
Immigrant	Girls	296	1.17	0.90–1.54	1.19	0.89–1.60	1.37*	1.01–1.85
	Boys	265	1.46**	1.11–1.92	1.46*	1.07–2.00	1.29	0.94–1.79
	Total	561	1.31**	1.08–1.58	1.30*	1.05–1.62	1.33*	1.07–1.66
Both parents	Girls	97	0.96	0.63–1.47	1.67*	1.07–2.60	2.12***	1.36–3.31
	Boys	74	1.16	0.72–1.87	1.59	0.93–2.70	1.20	0.68–2.12
	Total	171	1.05	0.76–1.44	1.66**	1.18–2.32	1.70**	1.20–2.40
Only mother	Girls	95	0.82	0.53–1.25	0.73	0.43–1.24	0.95	0.56–1.61
	Boys	105	1.18	0.79–1.77	1.26	0.78–2.02	1.46	0.92–2.33
	Total	200	1.00	0.74–1.34	0.97	0.68–1.37	1.19	0.84–1.68
Only father	Girls	104	2.08**	1.32–3.26	1.27	0.81–2.00	1.17	0.73–1.89
	Boys	86	2.35***	1.47–3.74	1.63	0.99–2.67	1.18	0.70–2.00
	Total	190	2.19***	1.59–3.03	1.39*	1.00–1.94	1.18	0.83–1.67

Values given in bold are statistically significant; Ref.: reference category.

^a^Dietary score based on compliance with Nordic Nutrition Recommendation for intake of carbohydrate, fat, protein, vitamins, minerals, and fibre.

^b^No participation or less than once per week in any organized activity.

^c^Age- and sex-adjusted BMI corresponding to adult BMI ≥ 25 kg/m^2^.

^d^It is adjusted for parental level of education (≤9, 10–12 and >12 years) and mutually adjusted for dietary score, low physical activity, and overweight.

OR and 95% CI were significant: *P < 0.05, **P < 0.01, and ***P < 0.001.

**Table 6:** Odds ratio (OR) and 95% confidence interval (CI) of dietary score, low physical activity, and overweight, by parental birth region.

Parental birth region	*n*	Dietary score^a^		Low physical activity^b^		Overweight^c^	
Father-mother		OR^d^	95% CI	OR^d^	95% CI	OR^d^	95% CI
Sweden-Sweden (Ref.)	2028	1.00		1.00		1.00	
Africa-Africa	16	1.80	0.64–5.02	0.49	0.11–2.17	0.90	0.25–3.17
Asia-Asia	59	0.95	0.56–1.60	2.19**	1.28–3.74	2.99***	1.76–5.07
Iran	24	0.83	0.37–1.87	1.07	0.42–2.74	3.38**	1.50–7.64
Turkey	5	0.52	0.08–3.20	0.67	0.07–6.06	16.24*	1.79–147.29
Europe-Europe (excluding Sweden)	48	1.24	0.69–2.23	1.27	0.67–2.40	1.22	0.63–2.38
Finland	21	1.63	0.64–4.00	1.85	0.75–4.53	1.75	0.70–4.41
Poland	9	1.07	0.28–4.03	0.98	0.20–4.74	1.13	0.23–5.48
Latin America-Latin America	7	0.61	0.13–2.78	2.21	0.48–10.08	9.66**	1.86–50.18
Mixed (excluding Sweden)	34	1.03	0.52–2.04	2.17*	1.07–4.39	0.83	0.34–2.01
Mixed (including Sweden)	390	1.44**	1.15–1.80	1.16	0.90–1.50	1.18	0.91–1.53
Unknown	7	0.38	0.07–1.95	1.29	0.25–6.75	0.67	0.08–5.62

Values given in bold are statistically significant; Ref.: reference category.

^a^Dietary score based on compliance with Nordic Nutrition Recommendation for intake of carbohydrate, fat, protein, vitamins, minerals, and fibre.

^b^No participation or less than once per week in any organized activity.

^c^Age- and sex-adjusted BMI corresponding to adult BMI ≥ 25 kg/m^2^.

^d^It is adjusted for sex, parental education level (≤9, 10–12 and >12 years) and mutually adjusted for dietary score, low physical activity, and overweight.

OR and 95% CI were significant: *P < 0.05, **P < 0.01, and ***P < 0.001.
